# Understanding Challenges Women Face in Flood-Affected Areas to Access Sexual and Reproductive Health Services: A Rapid Assessment from a Disaster-Torn Pakistan

**DOI:** 10.1155/2024/1113634

**Published:** 2024-04-01

**Authors:** Mariam Ashraf, Sara Shahzad, Pamela Sequeria, Anam Bashir, Syed Khurram Azmat

**Affiliations:** ^1^Integrity, Islamabad, Pakistan; ^2^Department of Public Health and Primary Care, University of Cambridge, Cambridge CB2 1TN, UK; ^3^London School of Economics and Political Science, London WC2A 2AE, UK; ^4^APPNA Institute of Public Health, Jinnah Sindh Medical University, Karachi, Sindh, Pakistan; ^5^Department of Technical Services, Marie Stopes International, Karachi, Sindh, Pakistan

## Abstract

**Introduction:**

According to the Global Climate Risk Index, Pakistan is ranked as the fifth-most vulnerable country to climate change. Most recently, during June-August 2022, heavy torrential rains coupled with riverine, urban, and flash flooding led to an unprecedented disaster in Pakistan. Around thirty-three million people were affected by the floods. More than 2 million houses were damaged, leaving approximately 8 million displaced and approximately 600,000 people in relief camps. Among those, 8.2 million women and 16 million children are the worst affected, with many requiring urgent medical and reproductive healthcare. To plan an efficient healthcare program and a climate-resilient health system, it is crucial to understand the issues that the affected people face during floods. *Methodology*. This rapid assessment included the population in the most severely affected districts across the four provinces of Pakistan. A mixed methods approach using qualitative and quantitative techniques was utilized. A total of 52 qualitative, in-depth interviews were conducted with community-level healthcare providers, national and provincial government departments, and development partners involved in relief activities. Using a structured questionnaire, the quantitative cross-sectional survey was conducted with a final sample of 422 women, married and unmarried (15-49 years old), residing in the relief camps in the flood-affected areas. The outcome variable of the survey was the access to sexual and reproductive health services faced by the women in the flood-affected districts. Data collection took place four months postfloods during Nov-Dec 2022, while the data analysis was conducted between Dec 2022 and Jan 2023. The quantitative data was analyzed using SPSS (Statistical Package for the Social Sciences) version 20, and qualitative data was analyzed using NVivo 12. Ethical consent was sought from all the participants. Ethical approval was also sought from the ethics committee of the Health Services Academy, Government of Pakistan.

**Results:**

The findings indicated that, overall, all the provinces were unprepared for a calamity of such a large magnitude. Access to services and health data reporting from the flood-affected areas was challenging mainly due to a shortage of trained health workforce because of the displacement of a large volume of the health workforce. Overall, equipment, medicines, supplies, and food were scarce. Women residing in the camps were markedly affected, and 84% (375) were not satisfied with the flood relief services provided to them. The floods impacted their monthly income as 30% (132) of respondents started depending on charity postfloods. Almost 77% (344) reported limited access to sexual and reproductive health services and had yet to receive sanitary, hygiene, and delivery kits, while 69% (107 out of 154) of girls stopped schooling postfloods. Almost 77% (112) of the married women reported having a child less than one year of age. Yet, only 30% (44 out of 144 currently married women) were using any form of family planning method—damage to the health facilities affected access to overall maternal care services.

**Conclusion:**

The findings concluded that there was no planning for sexual and reproductive health services in the flood-affected areas. Several barriers were identified. The government and development partners needed to prepare to cater to women's needs during the floods. The findings highlight the need for collaborative efforts between the government, civil society, and development partners to address the challenges faced in disaster management and strengthen disaster management capacity.

## 1. Introduction

Pakistan faces some of the highest disaster risk levels in the world, ranked 18 out of 191 countries by the 2019 Inform Risk Index. This risk is driven particularly by the nation's exposure to natural disasters and the risks of internal conflict. However, Pakistan also has high exposure to flooding, including riverine, flash, and coastal, as well as some exposure to tropical cyclones and their associated hazards, and drought [1]. As a result of these, Pakistan is ranked as the eighth country in the world that is most vulnerable to long-term climate risk [2].

Between June and August 2022, torrential monsoon rains and a combination of riverine, urban, and flash flooding led to an unprecedented disaster in Pakistan. Based on recent data, floods in Pakistan have left over 1,700 dead and hundreds of thousands injured, and about 33 million estimated people (one in seven) have been affected—over a million houses are totally or partially damaged, leaving behind millions needing urgent shelter (approximately 8 million people displaced and around 600,000 people living in relief camps) [3, 4]. The disaster has also disproportionately affected women (8.2 million) and children (about 16 million), and they are the worst affected, with many urgently requiring medical and reproductive health services [5]. Flooding has also severely damaged Pakistan's already crumbling healthcare infrastructure, with an estimated 10 per cent of the health facilities damaged [6]. It has also destroyed some vital infrastructures, including nearly 27,000 schools [6]. Unfortunately, rainwater is still stagnant in places putting millions at increased risk of diseases such as cholera, malaria, dengue, and skin infections [7]. Many of the hardest-hit areas are among the most vulnerable in Pakistan, where the population already suffers high malnutrition rates and poor access to water and sanitation [7].

Climate change serves as a threat multiplier by which existing community-level health disparities, antiquated critical infrastructures for transportation, water, food distribution, and communication, as well as other vulnerabilities related to social and environmental determinants of health (e.g., inequities related to poverty, housing, stock, access to healthcare, food security), are amplified, accelerated, or worsened and typically cooccur in the event of a weather-related disaster [8].

According to some of the literature, female survivors of climate-related disasters are more likely to face decreased life expectancy, mental health disorders, exploitation and risk for trafficking, and increased complications in childbirth. During floods, the challenges faced by women and girls, pertaining to their sexual and reproductive health (SRH) needs, are magnified. Given the lack of space, facilities, and necessities, women cannot manage monthly menstruation in a safe, private, and dignified manner. Additionally, in crises, one in every five women of childbearing age is likely to be pregnant. The risk of fatality and labour complications seems to intensify during this time due to limited healthcare services; other challenges include limited or no use of private and public toilets (since many are destroyed or submerged in water), rampant open defecation, and compromised personal hygiene [9]. Disasters also exacerbate gender-based violence, including sexual exploitation and abuse [10]. Awareness-raising, education, and the issue of warnings appear to be key initiatives to mitigate or prevent flood morbidity and mortality, especially among people living in low- and middle-income countries [11].

Other studies also indicate that exposure to floods during pregnancy has long-term negative effects on the cognitive development of children. Greater maternal support and public health interventions during pregnancy and early life after a natural disaster are warranted to facilitate healthy cognitive development in later life [12].

Disasters are not discriminatory but affect everyone irrespective of age, gender, religion, or any other determinant. However, women tend to experience the greater brunt of disaster consequences. Women are more vulnerable than men to the effects of natural disasters and climate change, not only because of biological and physiological differences but also because of socioeconomic differences and inequitable power relations. Natural disasters and climate change often exacerbate existing inequalities and discrimination, including gender-based ones, and can lead to new forms of discrimination [13]. During emergencies, routine behaviours are altered drastically. Women who use contraception may not have access to contraceptive drugs or devices or may forget to take or use them, risking unplanned pregnancies and sexually transmitted diseases. In addition, delivery of prenatal care and delivery becomes challenging as the health system is overstretched risking pregnancy complications and childbirth in unsafe conditions increasing maternal and infant morbidity and mortality [14].

## 2. Rationale

In the Pakistan context, little research has been conducted on the impact of floods on health systems and the healthcare needs of women. To fill this gap, the current study was undertaken to understand the health challenges encountered by women during floods and identify key gaps and barriers women had to face in accessing SRH services in the flood-affected districts of Sindh, Punjab, KP, and Baluchistan provinces in Pakistan.

The research was undertaken by Integrity and supported by the British High Commission-funded Delivering Accelerated Family Planning in Pakistan (DAFPAK) programme and will also provide an in-depth understanding of the challenges faced by government partners and private-sector organizations in response to such catastrophes. Identifying the health system challenges in disasters can lead to improving the systems, which will in turn help to be effectively prepared for future destructive disasters.

### 2.1. Study Research Objectives and Questions

There were three study objectives and three research questions that this study seeks to answer (refer to Table 1). The first two questions were assessed through qualitative information, and the third one was assessed through a quantitative survey.

## 3. Materials and Methods

### 3.1. Study Design, Participants, and Sampling Strategy

**Figure 1:**
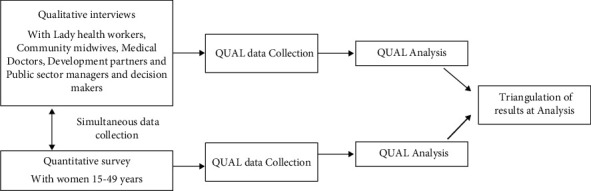
Schematic figure showing mix-method approaches.

The research study used a mixed methods design using the qualitative and quantitative data collection approaches. The most common and well-known approach to mixing methods is the triangulation design. The purpose of this design is “to obtain different but complementary data on the same topic to best understand the research problem.” This design intends to combine quantitative methods' differing strengths and nonoverlapping weaknesses. Triangulation design is a one-phase design in which quantitative and qualitative methods are implemented during the same timeframe. This design's single-phase timing is why it has also been called the “concurrent triangulation design.” It generally involves the concurrent but separate collection and analysis of quantitative and qualitative data to understand the research problem best. The convergence model (Figure 1) is used in this research and represents the traditional mixed methods triangulation design model. In this model, quantitative and qualitative data are collected and analyzed separately on the same phenomenon, and then, the different results are converged (by comparing and contrasting the different results) during the interpretation.

Kindly refer to Table 2 for the summary of quantitative and qualitative data collection method and sample size.

The quantitative component comprised a cross-sectional survey conducted with women of reproductive age (15-49 years old) residing in the relief camps in flood-affected areas utilizing a structured questionnaire. The sample included the population in the most severely affected district across the four provinces of Pakistan. According to the National Disaster Management Authority (NDMA) [15], the total population affected by floods in the four provinces is estimated to be 32,941,129, approximately 15% of the total population of Pakistan. The total number of women affected during these floods is estimated to be 12,186,374 (36%), including 25% of currently married women of reproductive age (CMWRA), 10% of adolescent girls aged 10-19 years, and 2% of currently pregnant women. Considering the above estimates, a sample size of 359 was calculated using EPI info stat calc for the survey. This calculation was done considering a 5% significance level, 36% of the population affected by flood, and considering a nonresponse rate of 10%; the final total derived sample was 422.

The qualitative component comprised of interviews with all available front-line healthcare workers at the time of the survey, who were providing services in the catchment area of the relief camps located in the flood-affected districts. For example, around 13 lady health workers (LHWs), community midwives (CMWs), and 09 medical officers were interviewed to identify the key barriers and challenges in providing services to women. In addition, 12 women who had recently given birth in the relief camps within the last three months were also interviewed to gather information on barriers and access to services. Another 29 interviews were conducted with district, provincial, and national government authorities and international development partners (those who provided consent and were selected purposively based on their presence in the flood-affected districts), to assess their response to the floods, catering to the needs of women and children, and how efforts were made to ensure safety for the population and preparedness plans for the current scenario and in the event of future climate-induced disasters. The qualitative sample for this study was carefully selected to ensure the representation and understanding of flood relief activities in the relevant provinces and districts. All participants of the qualitative interview provided written consent. Earlier, a mapping list was created to cover all provinces and districts affected by this disaster, and interviews were then conducted. The most relevant and knowledgeable individuals from government and development partners were identified and interviewed. This approach allowed the researchers to sample the target audience who has an in-depth understanding of the flood relief activities in the area to gauge how the maternal and child health services worked during a disaster of this scale and see the impact of this work.

### 3.2. Survey Geography

From each province, district selection was based on (a) the severity of the impact of the floods (populations most affected by floods that include displaced populations, damage to roads, schools, health facilities, and homes) and (b) the proportion of displaced populations living in camps. Three districts, Khairpur, Sanghar, and Badin from Sindh, Rajanpur from Punjab, Dera Ismail Khan (DI. Khan) from Khyber Pakhtunkhwa, and Jaffarabad from Baluchistan, were selected. From each district, the most affected union council (UC) was selected based on the list from the district disaster management authority, specifically focusing on the population living in camps. From each UC, the largest government-placed camp was selected to collect a sample of seventy women that were selected systematically with a random start. The sample was equally distributed in the selected districts.

### 3.3. Inclusion and Exclusion Criteria for Qualitative and Quantitative Survey

- Respondents for the qualitative interviews were those who were directly providing health services, financing, or managing flood response activities
- All those who consented to participate in the research study were interviewed
- Adolescent girls of 10-15 years of age were interviewed who provided assent, and then, consent was taken from at least one of the parents

Based on the above criteria, the response rate was 100% with no refusals for the quantitative component.

### 3.4. Qualitative Guidelines/Quantitative Survey Instrument

For qualitative data, open-ended guidelines were developed considering the challenges faced during floods. For each stakeholder, separate guidelines were developed.

The quantitative survey tool of data collection was adapted from previously tested survey tools pertinent to the flood situation [16]. The adapted tool was then pilot tested on women residing in the camps of flood-affected areas other than the selected sites. The face validity of the survey instrument was tested during the pilot phase, whereas the factor analysis technique was used to validate the questionnaire. The questionnaire reliability was measured through Cronbach's alpha test for the questions representing the effect of flood. The Cronbach's alpha for all the questions was 0.83, indicating excellent response consistency.

The survey instrument was initially finalized in English. The instrument's English version was translated into the local language, i.e., Urdu, by an independent person (fluent in Urdu translation) who was not directly involved in this research project. The final versions were then back-translated into English by a second independent person (fluent in English translation). The reverse-translated version was compared and reviewed for consistency with the original English version of the instrument. Any inconsistencies found through this comparison were rectified between the instrument's reverse-translated and Urdu versions.

Data variables for the survey included the dependent variable, which was the access to SRH services by the women, and independent variables, which included age, marital status, education status, monthly income, current residence, and relocation after the floods.

### 3.5. Data Management, Collection, and Analysis

The data collection process for this study was planned and executed with the support of district health and disaster management authorities. Both qualitative and quantitative data were collected from women residing in government-provided camps in flood-affected districts. Field researchers with experience in both types of data collection and data entry were recruited and trained, and a quality assurance officer was deployed to maintain data quality. The quantitative data was cleaned and validated using Excel and SPSS version 19, and various statistical analyses were conducted to identify trends and patterns. For qualitative data, interview notes were documented, and the interviews were recorded where possible and transcribed verbatim. NVivo software was used for content analysis, and data triangulation was employed to ensure validity and reliability. The application of this approach to data collection and analysis enhances the credibility and robustness of the study's findings while the triangulation was ensured as the data was gathered from different people in different hierarchies and working on different designations.

### 3.6. Ethical Considerations

All participants were informed of the research aims and objectives. Informed consent was sought from all the participants. This was done as verbal consent and where possible written consent was sort to ensure that all participants fully understand that the data collection is only for research.

The security of collected information and protocols to ensure anonymity and confidentiality was followed. No names or identifiable details were used, and the participants were deidentified in the final dataset for anonymity. All participants were told that they had the right to refuse/stop further participation at any given point, without providing a reason. In the case of a minor, while interviewing, the adolescent girl's permission/consent was also sought from the parents.

Ethical approval was also sought from the ethics committee of the Health Services Academy, Government of Pakistan No. 7-82/IERC-HSA/2022-44 on 1st November 2022.

## 4. Results

The findings are shared according to the research questions already shared earlier in Table 1.

### 4.1. Qualitative Findings

Triangulated findings RQ1: To what extent the overall health system was prepared for managing and responding to the flood situation specifically for reaching vulnerable groups (women and girls)?

1. Floods deteriorated the already weak existing healthcare system
2. Despite accessibility issues caused due to floods, the Department of Health and Integrated Reproductive Maternal and Child Health (IRMNCH) programmes in collaboration with government departments and development partners in all provinces provided immediate support to pregnant women, children, and CMWRAs and organized medical camps in collaboration with development partners
3. Data reporting from the flood-affected areas was also considered to be a challenge in the initial phase of the catastrophe, especially in KP and Baluchistan owing to far-flung areas and the inadequate presence of trained human resources
4. There was a need for an early warning system or preparedness; however, some provinces were better prepared to provide an immediate response. The Provincial Disaster Management Authority (PDMA) in Punjab mobilised and provided immediate relief compared to other provinces
5. Shortage of medicines, supplies, and service delivery professionals was reported across the provinces
6. There was a lack of adequate financing to cater to the needs of the affected populations, especially in Sindh and Baluchistan

At the national level, a premonsoon warning was generated by the National Disaster Management Authority (NDMA) on June 15, 2022. As a result, disaster preparedness-related activities were started since the generation of this alert. However, a calamity of such magnitude was not expected. Flood cells were formed in all districts by June, and as soon as the warning was issued, they became active 24/7. In Sindh, risk monitoring and early warning were delayed. Initially, tents were provided to the affected population, which could have been more effective, and later, 14 tent cities were established. In Baluchistan and KP, early warning from NDMA/PDMA was received, and a response was generated with the support of partners.

In response to floods, at the national level, the National Flood Response and Coordination Centre (NFRCC), with representatives from federal and provincial governments and armed forces, was developed to better articulate and support flood relief efforts during the rescue, relief, and rehabilitation/reconstruction activities. However, all provinces were unprepared for a calamity of such a large extent. The disaster management authorities at national, provincial, and district levels each year prepare contingency plans to manage disasters related to monsoon floods, but they fail to respond adequately to the disaster to this extent.

In the worst-case scenario, we were informed that 62000 HH would be affected, and we had 82 tents. But the disaster became too much. The rainfall received was too much in some of the districts and it was unprecedented rainfall that resulted in a catastrophe of this extent. (PDMA Sindh)

This was 10 times more than expected. The flood water from neighbouring provinces like Sindh and Punjab washed down to Naseerabad division (Baluchistan) and we still have 7-8 ft of water standing in this division. (Head of the committee leading flood response in Baluchistan)

#### 4.1.1. Challenges of Data Reporting

Data reporting from the flood-affected areas was considered a challenge in the initial phases of the catastrophe. Misdiagnosis of emergency illnesses was reported. The inability to diagnose disease symptoms, specifically during the disaster, was a major concern. Registration of pregnant women was also found to be challenging. The human resources available could not record and register the data of the affected population for submission to the higher management tiers to enable them to respond accordingly.

We were not able to receive comprehensive data regarding the situation of the population. We try to get as much information as can and specifically with a focus on MNCH services. (Coordination Officer Floods-MoNHSRC)

#### 4.1.2. Lack of Medicines, Supplies, and Food

Stakeholders operating in flood-affected regions claimed that the residents living in shelters required medicines, food, and other supplies because of the floods.

Even though many government and non-governmental organizations are attempting to provide them with such facilities, they cannot meet the people's demands owing to a shortage of medicines and food. There was also a shortage of medicine for pregnant women. (Head of the committee leading flood response in Baluchistan)

It was reported that there was a shortage of equipment and service delivery even before the floods.

Floods deteriorated the already weak existing health care system. (WHO representative)

Lack of food supplies was also one of the greatest challenges for the affected population.

Food crisis had a significant impact on women and children. Due to a scarcity of food, there was a risk of malnutrition among the children living in tents or without shelter and who lacked the financial means to purchase food or medicine. (Lady health worker)

#### 4.1.3. Access Issues due to Infrastructure Damage

Infrastructure damage was reported along with damage to roads, houses, and health facilities. All this added to the difficulty of accessing the affected population. Accessibility issues in some affected areas especially in KPK, Sindh, and Baluchistan were reported. In Kalam and Swat from KPK and Turbat, Baluchistan, there was a delay in providing food and other supplies due to land sliding and erosion of land routes/road network.

Due to flooding, ambulances could not access the affected area and the patients could not reach the hospital due to the destruction of the infrastructure. (Head of the committee leading flood response in Baluchistan)

#### 4.1.4. Shortage of Health Workforce

There was a shortage of healthcare professionals due to the displacement of a large volume of the health workforce. Development partners provided additional human resources to medical camps and health facilities. Training of the medical staff in disaster management was reported to be a crucial aspect in managing the humanitarian response. Specifically, from Baluchistan, it was shared that there is a need for training in disaster management.

There was a shortage of community-based lady health workers (LHWs) in Sindh and Balochistan as they were also displaced by the floods with damage to their health houses. This created a huge gap as LHWs provide support at the community and household levels. They also provide the required information on pregnant and lactating women and children.

Triangulated findings RQ2: How effective were coordination mechanisms to respond to the floods at the district, provincial, and national levels in ensuring the availability of services?

Overall, there appeared to be good district, provincial, and national coordination among various government departments.

1. UN organizations (WHO, UNICEF, and UNFPA) actively established coordination mechanisms
2. Issues of lack of coordination were identified in Sindh between health and population welfare departments and between the DG office and the Integrated Reproductive Maternal Newborn and Child Health (IRMNCH) Programme
3. DAFPAK partners identified stronger coordination at district levels with government departments of health and population welfare; however, in Punjab as MSS is acquiring NOC, there was a lack of coordination at the provincial level
4. The UN agencies developed a joint operation framework, with UNOCHA coordinating with all NGOs based on lessons learnt from the COVID-19 response

#### 4.1.5. National and Provincial Level Coordination

The lead for flood coordination at the national level stated that “along with the Ministry of Health and other departments, they worked in coordination. First, we got funding from UNICEF, WHO, UNFPA, WFP, and UNDP; everybody was on board and helped in their respective districts”.

IRMNCH Punjab mentioned the active involvement of UNICEF, WHO, and UNFPA to support in providing maternal and child health services. “Our primary focus was to eliminate duplication among partners.”

IRMNCH Sindh reported a lack of coordination with the population welfare department. They also mentioned that “while the primary responsibility to provide care for pregnant women, CMWRAs, and adolescent girls affected by floods was under the domain of the IRMNCH Programme, development partners response was provided to the Director General (DG) Health's office.”

The Department of Health in Khyber Pakhtunkhwa's flood response unit and integrated health project (IHP) leads stated effective response and coordination by UNFPA, WHO, UNICEF, and UNFPA supported with providing additional healthcare professionals. “Flood-affected women were provided with MNCH services and support, the WHO supported by providing hygiene kits, while UNICEF ensured availability of baby and delivery kits.”

Owing to capacity and resource availability in Baluchistan province, it was encouraging to note the interprovincial support that was provided by the Punjab and Sindh provinces in the form of human resources and medicines. The Deputy Director of the MNCH Programme in Baluchistan mentioned effective coordination with the UN partners in establishing medical camps.

#### 4.1.6. District-Level Coordination

The district-level coordination was more effective while the development partners also indicated greater coordination at the district level with the involvement of the district administration, which was coordinating with all government departments and development partners.The district health officer of all the target districts reported smooth coordination with local organizations that supported the establishment of a safe shelter space for pregnant women, who were provided food and delivery services. Based on the learning from the COVID-19 response factored in the flood response, a UN joint operational framework was developed following the 4Rs framework (reduction, readiness, response, and recovery) for the highly affected districts. UNICEF, UNFPA, and WHO focused on providing health and family planning services.

### 4.2. Quantitative Findings

Triangulated findings RQ3: What are the key barriers faced by the women to accessing healthcare services in general and family planning (FP) services in specific during the floods?

1. Accessibility to health facilities in terms of transportation and financial limitations was a major barrier for women
2. 77% (344) reported limited access to SRH services and had not received sanitary, hygiene, and delivery kits
3. 95% (425) had limited access to separate latrines and bathing facilities at the camps, mainly because only one latrine was available in the camp facilities
4. Two-thirds (-67% (299)) reported using the available public facility or government-organized camp for seeking healthcare services
5. Anaemia, diarrhoea, fever, and skin infections were the most common healthcare problems faced by women
6. The key barriers in seeking health services were reported to be distance (44% (198)), security (25% (113)), and followed by affordability and nonavailability of female staff (15% (68))
7. 69% (107 out of 154) of girls stopped schooling in the postflood scenario, mainly because they were displaced (49% (76))

#### 4.2.1. Sociodemographic Characteristics of the Cross-Sectional Survey Respondents

Most of the respondents, 57%, were between 15 and 25 years of age. Two-thirds or approximately 65% (292) were married, and nearly three-quarters or 76% of them (340) had no formal education. 79% (353) relocated due to floods, and currently, 76% (340) were residing in shelters/camps, out of which 30% (132) of the respondents were dependent on charity (Table 3).

#### 4.2.2. Experience during Floods

None of the participants reported experiencing any violence during floods. However, 80% (358) shared that in case of any violence, the female survivor will seek help from their mother. The most common site of violence was reported to be at home (43% (192)) followed by travelling alone (35% (157)). Approximately 77% (344) reported limited access to SRH services and had not received sanitary, hygiene, and delivery kits. The vast majority, 95% (425), had limited access to separate latrines and bathing facilities at the camps mainly because only one latrine was available in the camp facilities. About two-thirds or 67% (299) reported using the available public facility or government-organized camp for seeking healthcare services. Anaemia, diarrhoea, fever, and skin infections were the most common healthcare problems faced by women. The key barriers in seeking health services were reported to be distance (44% (198)), security (25% (113)), and followed by affordability and nonavailability of female staff (15% (68)) each, respectively. Nearly all respondents or approximately 96% (428) shared that their health deteriorated due to floods, while only 4% (19) shared that their health had improved. Overall, the majority, 84% (375), were satisfied with the services that were provided in the camps during the floods (Table 4).

#### 4.2.3. Schooling of Adolescent Girls

More than two-thirds i.e. 69% (107) of girls stopped schooling in the postflood scenario, mainly because they were displaced (71% (76)), parents did not give permission (20% (21)), and schools had been destroyed during floods as reported by 9% (10) of the girls (Table 5).

#### 4.2.4. Uptake of Family Planning (FP) Methods

Over 77% (112) of the CMWRA reported having a child less than 1 year of age, yet only 30% (44) reported using any form of FP method, indicating a need to counsel on birth spacing. 70% (100) of the CMWRA reported not using any method. The most common method of family planning used by women was oral contraceptive pills (41%), followed by injectables (36%), emergency contraceptive pills (14%), followed by implants (5%), and IUCD 2% (refer to Table 6). Of the 100 respondents who were not using any contraceptive method, 35% of the respondents mentioned religious reasons, 17% faced opposition from family, 7% did not know of any method, 4% cited a lack of supplies, and 1% each mentioned fear of side effects, lack of knowledge of the source, and access/distance issues (Table 6).

#### 4.2.5. Challenges Faced by Pregnant Women

Almost 18% of the women were primigravida, while about one-third (34%) shared that the age of the youngest child was up to 1 year. The minimum age reported was 11 months. 27% (40) reported the age of the youngest child between 1 and 2 years. This indicates that the awareness level pertinent to spacing and using FP methods was minimal among pregnant women. Less than half (44%) of the currently pregnant women were receiving antenatal care services from public sector/government facilities, followed by 25% from midwives and 14% from family members. Damage to the health facilities had affected access to ANC services for pregnant women as most preferred availing services from a government facility. About two-thirds or 65% (104) of currently pregnant women planned to give birth at a public facility/hospital, while 13% were not decided, and 4% planned to give birth at home while 3% at a private facility. The preference for the public facility could be related to the cost. Nearly half or 46% (62) expected delivery costs up to PKR 5,000. More than half or 52% (77) of pregnant women shared that they are undecided about their future fertility preference. Approximately 28% (42) would not like more children, hence the need for adequate postpartum counselling and services (Table 7).

#### 4.2.6. Health Status Situation after Floods

To check the normal distribution, the Shapiro-Wilk test of normality was conducted to determine whether age, education, health status, marital status, and access to services data are normally distributed. The results indicate that we must reject the null hypothesis for normal distribution (*P* ≤ 0.001) and conclude that data is not normally distributed.

Therefore, we conducted the Fisher's exact test, a specialized version of the chi-square test, which is particularly useful when dealing with small samples or low expected cell frequencies. While both tests indicate significant differences, the Fisher's exact test provides more precise *P* values, especially in cases with small sample sizes or sparse data.

Women were asked about their health status postfloods, whether it deteriorated or remained unchanged. It was found that the health of married women deteriorated compared to unmarried, and this was found significant with a *P* ≤ 0.001. Similarly, the majority of the women, both less than or older than 25 years, also reported deterioration in health (*P* ≤ 0.001). Those living in shelters (75%) and having limited access to SRH services (95%) reported deterioration in their health postflood compared to their counterparts, but this was not statistically significant (*P* ≥ 0.05). Similarly, no significance was found when correlating education, income status, and current place of residence (Table 8).

## 5. Discussion

This research is aimed at gaining insights into the barriers faced by women in seeking SRH services during recent floods and the preparedness of the health system to respond to climate events. The current study highlights various challenges that arose after the floods, including a lack of preparedness; inadequate data reporting; shortages of medicines, supplies, and food; damage to infrastructure; and a shortage of healthcare workers. Despite the premonsoon warning generated by the National Disaster Management Authority (NDMA), the disaster management authorities were unprepared for a disaster of such magnitude, despite having contingency plans in place each year to manage disasters related to monsoon floods.

Stakeholders in flood-affected regions reported that residents living in shelters needed essential items, such as medicines, food, and other supplies. The lack of access to medicines, supplies, and food exacerbated the existing weaknesses of the healthcare system. Infrastructure damage, including roads, houses, and health facilities, made it difficult to reach the affected population, particularly in KPK, Sindh, and Balochistan. Similar findings were shared in a study from Bangladesh pertinent to floods that the main challenges at the facility level were a lack of services and a shortage of medicines, equipment, and trained health workers [17]. Shafiq et al. suggest that to avoid the shortage of medicines and supplies in such areas, the government must allocate resources towards stockpiling and distributing essential medications and supplies to disaster-affected regions [18].

Furthermore, a shortage of healthcare professionals due to the displacement of a large volume of the health workforce further exacerbated the situation. Although the NDMA/PDMA issued an early warning, and a response was generated with the support of partners, all provinces were unprepared for such a significant calamity, which was not expected. The lack of preparedness resulted in challenges with data reporting from the flood-affected areas, misdiagnosis of emergency illnesses, and difficulties in registering pregnant women. Timely and accurate data reporting is crucial in assessing the on-field situation and systematically creating necessary policies. A UNDRR report published in 2020 emphasized the importance of data reporting in disaster management [18]. To improve decision-making and response, the present study also suggests that data collection and analysis be integrated into disaster management systems. To improve decision-making and response, the study suggests that data collection and analysis be integrated into disaster management systems [19]. This study identifies the need for trained human resources. Similar findings were also found in a systemic review that shares that training may be seen as a direct way to develop the competencies of health workers to respond to health needs in humanitarian crises, but without an environment supportive of the use of this training, scarce aid resources may be wasted [20]. A key lesson shared in the literature that also applies here is that training should not be implemented as a stand-alone strategy without overall system improvement required for ongoing success [21].

The findings further identify that 77% (344) of women reported limited access to SRH services and had not received sanitary, hygiene, and delivery kits. Anaemia, diarrhoea, fever, and skin infections were the most common healthcare problems faced by women. The key barrier to seeking health services was reported to be distance, at 44% (198). Research studies suggest that a lack of access to SRH care is the leading cause of morbidity and mortality among displaced women and girls of reproductive age. Maternal and newborn health is threatened by displacement and interruption of health services. Societal instability, trauma, economic vulnerability, a lack of education and work opportunities, and the disruption of family planning and medical treatment and prevention services further exacerbate the situation and may result in further mortality and morbidity [22].

About pregnant women, the study findings indicate that damage to the public health facilities affected access to ANC services for pregnant women. About two-thirds, 65% (104), of currently pregnant women planned to give birth at a public facility/hospital. The literature identifies that infrastructure or lack of resources was a factor reported to impact access to ANC. Studies found that women who perceived operational and infrastructure problems in their community were deterred from accessing ANC and faced poorer health outcomes as a result [23, 24]. A study in Pakistan in 2011 highlighted that women who gave birth in relief camps during the flood had no skilled birth attendants present, used unhygienic birth stations, and had poor postnatal services which increased the risk of mortality [25].

Similarly, access to family planning services was also found to be limited as only 30% (44) reported using any form of the FP method. Several reasons have been highlighted that include religious inhibitions, nonavailability of method, and opposition from the family. Studies suggest that natural disasters have been shown to decrease access to family planning services either by the destruction of healthcare facilities and infrastructure [26]. Our findings further indicate that overall women face deterioration in their health when living in shelters. This can also be due to the fact that the overall support system for providing food, water, sanitation, and health services was lacking. Continued investment is needed to ensure access to the full spectrum of high-quality sexual and reproductive health care. The Guttmacher Lancet Commission recommends that a comprehensive package of essential sexual and reproductive health services, including contraception and safe abortion care, be included in national health systems [27]. This will further support the system and will ensure sustained supply during any natural disaster.

The findings of the study highlight the need for collaborative efforts between the government, civil society, and development partners to address the challenges faced in disaster management and strengthen disaster management capacity. Essential measures, such as training medical staff in disaster management and building the resilience of communities to cope with disasters, should be taken. Additionally, investing in training and deploying healthcare professionals to disaster-affected areas is crucial to ensure adequate healthcare for affected populations.

With over 230 million population, Pakistan has the highest rate of urbanization in the South Asian region, possibly rising to 50%, making the country more vulnerable to floods and other climate disasters [28]. The country witnessed a colossal number of human lives lost due to the recent deadly flooding due to intense monsoons and catastrophic heatwaves, destroying almost $15 billion in housing and infrastructure, which will cost even more to rebuild. Based on rapid population growth and migration, any high-risk country's ability to mitigate the devastation caused by climate-related disasters depends on employing demographic advances that can shape our cities and communities. A recent review has cited the importance of developing response plans prioritized for disaster-prone countries, for family planning and reproductive health services, especially since women and girls often lose access to these essential services when they are at their most vulnerable [29]. Hence, disasters and epidemics of such magnitude can further damage the already fragile health systems, diverting resources away from essential sexual and reproductive health (SRH) services, threatening supply chains, and adversely impacting access to health facilities.

## 6. Conclusion

There is a need for better preparedness and response to SRH needs during disasters, particularly for vulnerable populations. The government, development partners, and organizations performing humanitarian and relief work must come together to ensure that essential SRH services are available and accessible during and after disasters. In addition, efforts should be made to integrate SRH and family planning services into overall health systems to reach the demands of marginalized and hard-to-reach populations during such a natural calamity. This will require training and capacity building for healthcare workers focusing on addressing the specific needs of women and girls in humanitarian settings. Finally, a multisectoral approach is needed to address the SRH needs of women and girls, recognizing that SRH is a basic human right. This will require collaboration across sectors such as health, population, education, and social services to ensure that the needs of women and girls are prioritized and met in times of crisis. Overall, the findings highlight the urgent need for action to improve access to essential SRH services for vulnerable populations during and after disasters. This will require a coordinated effort from governments, development partners, and organizations performing humanitarian and relief work to ensure that the SRH needs of women and girls are met in times of crisis.

## 7. Limitations

Transportation difficulties in the affected areas were one limitation during data gathering. It is also important to mention that the survey focused on women residing in the camps with a limited sample; therefore, the survey findings cannot be generalizable. Moreover, the self-reported data by women and girls living in the camps might be subject to biases based on women's individual experiences. The estimates presented in Table 8 are not adjusted for potential confounders/covariates, and this is one limitation of the study since this activity is aimed at conducting a “rapid assessment” of the heavily devastated and disaster-torn geographies and immediately shares the descriptive findings with the government of Pakistan and its developmental partners. The primary reason is that this subgroup analysis was not the main objective of our study; instead, our purpose was to descriptively explore patterns in different population segments or among certain characteristics of study participants. Therefore, we did not employ any regression techniques. Nevertheless, we have included this point in the limitations section, noting that these estimates are not adjusted, and any inferences or interpretations should be cautiously made.

However, using a combination of both quantitative and qualitative tools in this mixed methods approach can be considered a strength of this study and helps to ensure that the results of a study are closer to reality. In addition, the study provides insights into barriers women and girls face in accessing sexual and reproductive health services during floods, identifies key areas for improvement, and provides recommendations.

## Figures and Tables

**Table 1:** Research objectives and questions.

Objectives 1. To understand the extent of preparation of the health system for managing and responding to the flood situation specifically for reaching vulnerable groups (women and girls) 2. To learn about the coordination mechanisms at the district, provincial, and national levels to respond to the floods for ensuring the availability of services 3. To assess the key barriers faced by the women to accessing healthcare services in general and family planning (FP) services in specific during the floods Based on the objectives, the following are the key questions: RQ 1. To what extent the overall health system was prepared for managing and responding to the flood situation specifically for reaching vulnerable groups (women and girls)? RQ 2. How effective were coordination mechanisms to respond to the floods at the district, provincial, and national levels in ensuring the availability of services? RQ3. What are the key barriers faced by the women to accessing healthcare services in general and family planning (FP) services in specific during the floods?

**Table 2:** Summary of data collection method and actual sample.

Data collection method	Sampling size
Quantitative	
Cross-sectional survey	A total of 447 women and girls were covered during the data collection. Adolescent girls 10-15 years old (154), currently married women of reproductive age (CMWRA) 15 to 49 years (144), and currently pregnant women 15-49 years (149)
Qualitative	
IDIs with community-level providers	13 CMWs/LHWs 09 medical officers working in the camps
District-level IDIs	12 IDIs with members from the District Disaster Management Authorities, district health office, and social welfare office
Provincial-level IDIs	10 IDIs with provincial government departments of health, population welfare, and provincial disaster management authorities in all four provinces
National-level IDIs	4 IDIs with the Ministry of National Health Services, Regulation and Coordination, representatives from WHO, and UNFPA
Key informant interviews	7 IDIs with development partners providing relief services

**Table 3:** Sociodemographic characteristics.

Category	No.	%
Type of respondents	447	100%
Adolescent girl (15-19 years)	154	34%
CMWRA	144	32%
CPW	149	33%
Respondents' age		
15-19	167	37%
20-25	91	20%
26-30	73	16%
31-35	41	9%
>35	75	17%
Marital status		
Not married	155	35%
Married	292	65%
Monthly income prefloods (PKR)		
Up to 20000	298	67%
20001-50000	80	18%
>50001	69	15%
Monthly income postfloods (PKR)		
Dependent on charity	132	30%
Up to 20000	254	57%
20001-50000	42	9%
More than 50001	19	4%
Relocation after floods		
No	94	21%
Yes	353	79%
Current residence		
Own house	107	24%
Camps, shelter/rented	340	76%
Education status		
No education	340	76%
Some informal education	20	4%
Primary (classes 1-5)	37	8%
Secondary (class 10)	46	10%
College or higher	4	1%

**Table 4:** Experience during floods.

	*N*	%
Female survivors of GBV seek help from		
Mother	358	80%
Father	28	6%
Husband	22	5%
Do not seek help	39	9%
Common sites for GBV		
In the home	192	43%
While travelling alone	157	35%
At schools/universities	52	12%
In tents/camps	45	10%
Received materials (sanitary kits, hygiene kits, delivery kits)		
No	344	77%
Yes	83	23%
State of health compared to before and after the floods		
Improved	19	4%
Deteriorated/remain the same	428	96%
Place for seeking healthcare services in the current flood situation		
Public facility/health camp	299	67%
Family members	110	25%
Do not seek services	21	5%
Private facility	17	4%
Easy access to latrines and bathing facilities		
No	425	95%
Yes	22	5%
Primary health problems experienced during floods		
Anaemia	133	30%
Diarrhoea	130	29%
Fever	116	26%
Skin infections	68	15%
Barriers faced by women for seeking health service		
Distance	198	44%
Security	113	25%
Affordability	68	15%
Nonavailability of female staff	68	15%
Overall satisfaction with the services available		
Not satisfied	375	84%
Satisfied	72	16%

**Table 5:** Findings from adolescent girls.

Adolescent girls currently schooling *N* = 154		
Yes	47	31%
Stopped schooling	107	69%
Reasons for stopping school *N* = 107		
Displaced	76	71%
Parents do not permit	21	20%
School destroyed	10	9%

**Table 6:** Findings from CMWRA.

CMWRA findings	*N*	%
Place of last delivery N-111		
At home with a trained provider	1	1%
At home (Dai)	88	79%
Public facility	9	8%
Private facility	13	12%
Total	111	100%
Age of youngest child *N* = 117		
1-6 months	84	72%
7 months to 1 year	28	24%
>1-2 years	5	4%
Total	117	100%
Current use of any FP method *N* = 144		
No	100	70%
Yes	44	30%
Type of method use (*N* = 44)		
COC pills	18	41%
Injectables	16	36%
EC pills	6	14%
Implants	2	5%
IUCDs (Cu-T or multiload)	1	2%
Female sterilization	1	2%
Reasons for not using any FP method		
Religious prohibition	35	35%
Family opposition	17	17%
Lactating mothers/amenorrhea	23	23%
Not available/fear of side effects	17	17%
Want to get pregnant	8	8%
	100	100%

**Table 7:** Findings from CPW (*N* = 149).

	*N*	%
Current trimester		
First trimester	31	21%
Second trimester	97	65%
Third trimester	21	14%
Seeking ANC services from		
Government facility	64	44%
Midwives	36	25%
Family member	21	14%
Dai (untrained provider)	13	9%
Private facility	7	5%
Health camp	9	6%
Planning to conduct delivery at		
Government facility	104	65%
Not decided	20	13%
At home (Dai)	7	4%
Private facility	5	3%
Age of youngest child		
First pregnancy	27	18%
Up to 1 year	51	34%
1-2 years	40	27%
2-3 years	18	12%
>3 years	13	8%
Future fertility preference		
Undecided	77	52%
Want to have more children	20	13%
No more children	42	28%
Want to get pregnant	8	8%

**Table 8:** Health status situation after floods (*N* = 447).

Characteristics	Frequency	Deteriorated		Improved		*χ* ^2^	*P* value	Fisher exact test
	*N*	No	%	No	%			
Marital status								
Never married	155	143	33%	12	63%	7.18	*P* ≤ 0.001^∗^	*P* ≤ 0.001^∗^
Married	292	285	67%	7	37%			
Age								
15-25 years	258	242	57%	16	84%	5.71	*P* ≤ 0.001^∗^	*P* ≤ 0.001^∗^
>26 years	189	186	43%	3	16%			
Current place of residence								
Camp/shelter	340	322	75%	18	95%	2.9	0.05	0.05
Own house	107	106	25%	1	5%			
Access to SRH services and materials								
No	344	326	95%	18	5%	3.5	0.06	0.09
Yes	103	102	24%	1	5%			
Education status of woman								
No education	338	322	75%	16	84%	5.70	0.37	0.58
Educated	109	106	25%	3	16%			
Income after floods								
Charity dependent	132	130	30%	2	11%	0.79	0.06	0.07
Have a monthly income	315	298	70%	17	89%			
Relocation after floods								
No	94	92	21%	2	11%	1.31	0.25	0.38
Yes	353	336	79%	17	89%			

^∗^ *P* value is significant (*P* ≤ 0.05).

## Data Availability

The first and second author has all the data of the study, which can be requested for further analysis and research.

## Supplementary Materials

Supplementary Materials Quantitative tool/s and qualitative guidelines.
