# Supplementary material for: Understanding Challenges Women Face in Flood-Affected Areas to Access Sexual and Reproductive Health Services: A Rapid Assessment from a Disaster-Torn Pakistan
Source: Biomed Res Int. 2024 Apr 1;2024:1113634. doi: 10.1155/2024/1113634 (PMC11001467; doi:10.1155/2024/1113634)
Supplement: Supplementary Materials — Quantitative tool/s and qualitative guidelines. [file 1113634.f1.docx]

**SUPPLEMENTARY FILES**

**QUANTITATIVE: Cross-sectional survey tool^[[1]](#footnote-2)^**

Understanding challenges women face in flood-affected areas to access sexual and reproductive health services: A rapid assessment from a disaster-torn Pakistan.

**Cross-sectional survey Questionnaire**

| ***Inclusion Criteria***  **Following women categories residing in camps**  **Adolescent girls= 15-19 years (after seeking consent from a parent)**  **Currently married women of reproductive age group (CMWRA 15-49 years)**  **Currently Pregnant Women (CPW)**    *SECTION I. Introduction*  My name is_______________. I work for _______________. We are conducting an assessment in this emergency in ______________. The purpose of this interview is to learn about health services in the humanitarian response and women’s experiences in accessing these services. During the interview, I will ask questions about your experiences in seeking health care during this flood emergency. The answers you provide will be used to inform the government and other relief organisations providing health services with the aim of addressing the challenges and improve the access and delivery of health services. Your name will not be included in any documents or presentations, but we may include the name of this location. There is no direct benefit to you from being in this study. If you are uncomfortable with any questions during this interview you are free to stop participating at any time. You can also choose not to answer any of the questions. Please stop me at any time during the interview if you have questions or concerns. ***Is it OK to get started******?***  *SECTION II. Preliminary Information* | |
| --- | --- |
| **Was informed consent obtained**  YES ________ (Proceed)  NO ________ (STOP! Thank the participant for their time and do not proceed | |
| **Date:** | **Location of interview :** |
| **Start time:** | **End time** |

| **SECTION III: QUESTIONS: General Information** | | | |
| --- | --- | --- | --- |
| **GI1** | **Province** |  | |
| **GI2** | **District Name** |  | |
| **GI3** | **City Name** |  | |
| **GI4** | **Camp location** |  | |
| **GI5** | **Respondent’s Name** |  | |
| **GI6** | **Respondent category** | 1. Adolescent girl, 2 CMWRA, 3 CPW | |
| **GI7** | **Respondent age (years)** |  |  |
| **GI8** | **Cell Number(Optional)** |  | NA Refused |
| **GI9** | **Complete address** | Camp .#: ___________________Street:__________________  Area: ___________________________________________  Nearby: ________________________________________  UC #: __________________________________________  Any other landmark: ______________________________ | |
| **GI10** | **Name of the Interviewer** |  | |
| **GI11** | **Date of the Interview** | ________________  DD / MM / YYYY | |
| **GI12** | **Name of Field Supervisor** |  | |
| **GI13** | **Form Status**  Moved this question at the end of every module) | - Interview Completed - Visit Rescheduled - Interview Incomplete - Refused - Other (Specify) | |
| **GI14** | **Which Partner's Catchment area is this?** | 1. DKT 2. MSS 3. PSI  a. HANDS  b. RSPN 4. Palladium  5. UNFPA  6. OTHERS (please specify)------------------------------------------ | |

| **DISPLACEMENT INFORMATION** | | |
| --- | --- | --- |
| DI1 | Where did you live before the flood? | 1. Province 2. District 3. Taluka 4. UC 5. Village |
| DI2 | Type of residence | 1. Own home 2. Rented home 3. Any other |
| DI3 | Did you relocated after floods | 0 No--------------Skip DI4  1 Yes |
| DI3-A | What were your reasons for relocating? | 1. Shelter 2. Economic reasons 3. Feeling of insecurity 4. Decision to move made by the head of household 5. Following family 6. Following other community members 7. Social pressure/stigma 8. House damaged 9. house destroyed 10. Other, specify: |
| DI4 | How long have you been living here? (in current place) | Months |
| DI5 | Type of shelter | 1. Shelter/Government camp 2. A tent in a camp 3. A prefabricated camp/shelter 4. Other: specify |
| DI6 | How many people were in your household before the floods? |  |
| DI7 | How many people are in your household since/after the floods? |  |
| DI8 | What effect have you felt on HH expenditures compared to before and after the flood?  Mutiple choices allowed | 1. Food: increased, decreased, same 2. Water: increased, decreased, same 3. general health: increased, decreased, same 4. maternal health; increased, decreased, same 5. Health-related to disabled members:   increased, decreased, same   1. clothes: increased, decreased, same 2. reconstruction: increased, decreased, same 3. education: increased, decreased, same 4. treatment for trauma or psychosocial needs: increased, decreased, same 5. Others ------------------------------------- 6. Not Applicable |
| DI9 | Monthly income of the HH before the floods | 1. Less than 20,000 2. 20,001-50,000 3. 50,001-70,000 4. 70,001-100,000 5. Greater than 100,000 6. Any other |
| D10 | Monthly income of the HH after the floods | 1. None – dependent on charity/support 2. Less than 20,000 3. 20,001-50,000 4. 50,001-70,000 5. 70,001-100,000 6. Greater than 100,000 7. Any other |
| DI11 | No. of disabled persons in the HH | 1. Total number  2. None ____________Go to D16 |
| DI12 | Type of disability |  |
| DI13 | No. of females disabled | 1. Total number Go to DI15  2. None ____________ |
| DI14 | Type of disability |  |
| DI15 | Is the respondednt disabled | 0 No ___________Go to DI16  1 Yes |
| DI15a | Do you have difficulty seeing, even if wearing glasses? | 1. No – no difficulty 2. Yes – some difficulty 3. Yes – a lot of difficulty 4. Cannot do at all |
| DI15b | Do you have difficulty hearing, even if using a hearing aid? | 1. No – no difficulty 2. Yes – some difficulty 3. Yes – a lot of difficulty 4. Cannot do at all |
| DI15c | Do you have difficulty walking or climbing steps? | 1. No – no difficulty 2. Yes – some difficulty 3. Yes – a lot of difficulty 4. Cannot do at all |
| DI15d | Do you have difficulty remembering or concentrating? | 1. No – no difficulty 2. Yes – some difficulty 3. Yes – a lot of difficulty 4. Cannot do at all |
| DI15e | Do you have difficulty with self-care such as washing all over or dressing? | 1. No – no difficulty 2. Yes – some difficulty 3. Yes – a lot of difficulty 4. Cannot do at all |
| DI15f | Using your usual (customary) language, do you have difficulty communicating, for example understanding or being understood? | 1. No – no difficulty 2. Yes – some difficulty 3. Yes – a lot of difficulty 4. Cannot do at all |
| DI16 | No. of pregnant women in HH | 1. Total number  2. None____________Go to DI17 |
| DI16A | If yes-the expected remaining month of delivery |  |
| DI17 | No. of lactating mothers in HH | 1. Total number  2. None |
| DI18 | No. of adolescent girls | 1. Total number  2. None |
|  | - For interviewing Adolescent Girls go to Module 1 - For interviewing CMWRA Go to Module 2 - For interviewieng CPW go to Module 3 | |

| **Module 1: Adolescent Girl (15-19 years)** | | |
| --- | --- | --- |
| AG1 | What is your highest level of education | 1. No education 2. Some informal education 3. Primary (Class 1-5) 4. Secondary (Class 10) 5. College or Higher |
| AG2 | How would you describe your state of health compared to before and after the flood? | 1. Increased 2. Decreased 3. Same 4. Other, specify |
| AG3 | What are women's and girls’ primary health problems at present? (can select 5 options)?  Do not read the list | 1. No Disease 2. Anaemia and other nutrition-related 3. ill health 4. Diarrhoea 5. Fever 6. Physical injuries 7. Disabilities 8. Bleeding 9. Respiratory problem 10. Skin infection 11. Skin rashes 12. STD/HIV 13. Mental health: anxiety, distress 14. Other, specify |
| AG4 | Who makes the decisions about women's and girls’ access to health care in the current  scenario? | 1. I don’t know 2. Myself 3. My mother 4. My father 5. Both of my parents 6. Other……………………… |
| AG5 | What are the two most significant obstacles to girl's ability to access health care in the present scenario? | 1. Distance to the nearest healthcare facility is too far 2. Security reasons 3. No female health staff 4. Personal health issues   (disabilities, injured, etc)   1. No transportation 2. Can’t afford it 3. Nearby health facility damaged and non functional 4. Other, specify: |
| AG6 | Where is the first place girls go to when they need health care in the current flood situation? | 1. Rural/community clinic 2. Health camps 3. Government clinic/public hospital 4. Midwives 5. Community centres 6. NGOs clinic 7. Drug store 8. Family 9. Traditional practitioner 10. I don’t seek healthcare 11. Private clinic /hospital 12. Other…………………………… |

| **FOOD AND NUTRITION, WATER, NON-FOOD ITEMS** | | |
| --- | --- | --- |
| AG7 | On average, how many meals do you have per day before the flood? | 1. 0 2. One 3. Two 4. Three 5. 5. Three or more |
| AG8 | On average, how many meals do you have per day after the flood? | 1. 0 2. One 3. Two 4. Three 5. Three or more |
| AG9 | If there is a food shortage in the house, whose food needs are prioritized? | 1. Mother 2. Father 3. Boy children 4. Girl children 5. Elderly women 6. Elderly men 7. Other, specify 8. Don’t know |
| AG10 | If there is a food shortage in the house, who is least likely to get food so that others can? | 1. Mother 2. Father 3. Boy children 4. Girl children 5. Elderly women 6. Elderly men 7. Other, specify 8. Don’t know |
| AG11 | Do you have access to latrines and bathing facilities? | 0 No  1 Yes  If No Explain : |
| AG12 | If yes, What type of latrine/bathing facility is available? |  |
| AG13 | What are the factors affecting access to domestic water use? | 1. High water price 2. Damage to the water networks 3. My community is not served by a   water network   1. Insufficient water supply, specify: 2. Other, specify: |
| AG14 | Who usually collects food items and water for the family? (can pick up To 2) | 1. Self 2. Mother 3. Father 4. Boy children 5. Girl children 6. Elderly women 7. Elderly men 8. Other, specify 9. Don’t know |
| AG15 | Do women and girls have easy and reliable access to sanitary materials/napkins? | 0 No  1 Yes  If No Explain : |
| AG16 | Do you feel safe and comfortable using the latrine/bathing facility? | 0 No  1 Yes  If No Explain : |
| AG17 | Did you received a hygiene kit during the floods | 0 No  1 Yes  If No Explain : |
| **EDUCATION** | | |
| AG18 | Did you stop going to school after the flood? | 0 No  1 Yes  If No Explain : |
| AG19 | What are the factors affecting  school attendance? | 1. Parents / family members permission 2. School Destroyed 3. Lack of school uniform/clothing 4. Displaced 5. Violence at school/university 6. Sexual harassment at school 7. No separate latrine available 8. Feeling of insecurity 9. Other, specify |
| **PROTECTION AND ACCESS TO JUSTICE** | | |
| AG20 | Main safety/security problems faced by women and girls | 1. Domestic violence specify 2. Sexual harassment 3. Being trafficked 4. Travelling alone long distances 5. Early marriage 6. Incest 7. Honour killings 8. Other, specify |
| AG21 | Have you faced any violence after floods | 0 No  1 Yes  If yes Explain : |
| AG22 | Where do female survivors of violence go for help? | 1. To their mothers 2. To their fathers 3. To other family members 4. Health Clinic 5. Private clinic 6. Legal aid 7. Community leaders 8. Friends 9. International/local NGOs 10. Community centres 11. Police 12. Other, specify 13. Don’t seek help |
| AG23 | Where are acts of violence against women and girls most likely to occur? | 1. In the home 2. At schools/universities 3. In tents 4. In the shelter 5. While using the bathing facility in   the shelter   1. While travelling alone 2. While at work outside the home 3. Other, specify: |
| **ACCESS TO INFORMATION AND ASSISTANCE, PARTICIPATION** | | |
| AG24 | Have you been informed of the relief and recovery assistance in a language or manner that you  understand? | 0 No  1 Yes |
| AG25 | How are most women on this site getting information? | 1. Husband/Male 2. Member 3. Female gathering 4. Radio 5. Mosque 6. announcement 7. TV 8. Healthcare   providers   1. Newspaper 2. Camp meetings 3. van drives 4. government representative 5. Other |
| AG26 | Is there an effective way that you or your community can resolve problems about your current situation, including any threats to your security and ensuring everyone receives aid equitably? | 0 No  1 Yes |
|  | **SUMMARY: Adolescent Girls** |  |
| AG27. | Are you satisfied with the service provided to you in this camp? | 1. Unsatisfied 2. Average 3. Satisfied 4. Totally Satisfied |
| AG28 | What are the top three **barriers** to an effective response to this emergency? |  |
| AG29 | What top three factors **facilitated** the response in this emergency? |  |
| AG30 | What suggestions do you have for improving implementation in this setting? Please think about preparedness, response, and recovery. | 1.  2.  3. |
| AG31 | We talked about a lot of things today; do you have any final comments or questions? |  |
| AG32 | **COMMENTS BY INTERVIEWER** |  |
|  | **Form status question moved here**  **End Time also moved here** |  |

| **Module 2: Currently Married Women of Reproductive age group (15-49 years)** | | | | | | | |
| --- | --- | --- | --- | --- | --- | --- | --- |
| CMWRA1 | | What is your highest level of education | 1. No education 2. Some informal education 3. Primary (Class 1-5) 4. Secondary (Class 10) 5. College or Higher | | | | |
| CMWRA2 | | What is the highest level of education of your husband | 1. No education 2. Some informal education 3. Primary (Class 1-5) 4. Secondary (Class 10) 5. College or Higher | | | | |
| CMWRA3 | | Duration of Marriage | __\|__ Months/years | | | | |
| CMWRA4 | | Do you have children  (Have you given any birth to a child) | 0 No (Incase of ‘No’ skip to CMWRA 8)  1 Yes | | | | |
| CMWRA5 | | How many of them are still alive and of them how many are sons and daughters?  (**Total number of living children)** | Sons __\|__  Daughters __\|__  Total _____ | | | | |
| CMWRA6 | | How old is your youngest child? | __\|__ Months/years | | | | |
| CMWRA7 | | Where was you last delivery conducted | 1. At home with a trained provider 2. At home – Dai 3. Public facility – specify 4. Private facility -specify 5. Anyother | | | | |
| CMWRA8 | | How would you describe your state of health compared to before and after the flood? | 1. Increased 2. Decreased 3. Same 4. Other, specify | | | | |
| CMWRA9 | | What are women's primary health problems at present? (can select 5 options)?  Do not read the list | 1. No Disease 2. Anaemia and other nutrition-related 3. ill health 4. Diarrhoea 5. Fever 6. Physical injuries 7. Disabilities 8. Bleeding 9. Miscarriage 10. Delivery related problem 11. Respiratory problem 12. Skin infection 13. Skin rashes 14. STD/HIV 15. Mental health: anxiety, distress 16. Other, specify | | | | |
| CMWRA10 | | Who makes the decisions about your access to health care in the current scenario? | 1. Myself 2. My husband 3. The husband and the wife together 4. My mother 5. My father 6. My mother-in-law 7. My father-in-law 8. Other……………………… | | | | |
| CMWRA11 | | What are the two most significant obstacles for women to access health care in the present scenario? | 1. Distance to the nearest healthcare facility is too far 2. Security reasons 3. No female health staff 4. Personal health issues (disabilities, injured, etc) 5. No transportation 6. Can’t afford it 7. Can’t take time off work 8. Can’t take time away from home 9. Child-care responsibilities 10. Objection by family 11. Objection by husband 12. Other, specify: | | | | |
| CMWRA12 | | Where is the first place women go to when they need health care in the current flood situation? | 1. Community health workers (LHWs, CMWs, others) 2. Rural/community clinic 3. Health camps 4. Government clinic/public hospital 5. Midwives 6. Community centres 7. NGOs clinic 8. Drug store 9. Family 10. Traditional practitioner 11. Private provider 12. Private clinic 13. Private hospital 14. I don’t seek healthcare 15. Other…………………………… | | | | |
| CMWRA13 | | Are you currently using any FP method? | 0 No ------------------Go to CMWRA 16  1 yes | | | | |
| CMWRA14 | | If yes duration of use | ______Months/years | | | | |
| CMWRA15 | | If yes, which method are you currently using? | 1. Female Sterilization 2. Male Sterilization 3. IUCDs 4. Implants 5. Injectables 6. COC pills 7. EC pills 8. Condoms 9. Lactational Amenorrhea 10. Standard days method 11. Rhythm Method 12. Withdrawal   Other (Specify)________ | | | | |
| CMWRA16 | | If No, can you tell us why you are not using any FP method to prevent pregnancy? | 1. Not having Sex 2. Infrequent Sex 3. Menopausal/Hysterectomy 4. Can’t get pregnant 5. Not Menstruated since last birth 6. Breastfeeding 7. Up to God/Fatalistic 8. Myself/husband opposed 9. In-laws opposed 10. Religious Prohibition 11. Knows no method 12. Knows no Source 13. Side effects/ Health concerns 14. Lack of access/ Too far 15. Costly 16. Preferred method not available 17. No method available 18. Inconvenient to use 19. Interferes with body’s normal Processes 20. Any other | | | | |
| CMWRA17 | | Were you using any method prior to floods | 0 No (Goto CMWRA 19 incase of ‘No’)  1 Yes | | | | |
| CMWRA18 | | If yes which method | 1. Female Sterilization 2. Male Sterilization 3. IUCDs 4. Implants 5. Injectables 6. COC pills 7. EC pills 8. Condoms 9. Lactational Amenorrhea 10. Standard days method 11. Rhythm Method 12. Withdrawal   Other (Specify)________ | | | | |
| CMWRA19 | | What sexual and reproductive health services have been available to you since these floods | 1. Information/counselling 2. Hygiene kits 3. Sanitary kits 4. Medicines/supplies 5. Contraceptives 6. Clean delivery kits | | | | |
| CMWRA20 | | Do you have access to activities and services to prevent unintended pregnancy available in this setting?  Prompt each service/activity.) | **Service/Activity** | | **No =0** | **Yes =1** | **Don’t know = 99** |
|  |  |  | 1a = Contraceptive pills | | 0 | 1 | 99 |
|  |  |  | 1b = Injectables | | 0 | 1 | 99 |
|  |  |  | 1c = Intrauterine device (IUD) | | 0 | 1 | 99 |
|  |  |  | 1d = Implant | | 0 | 1 | 99 |
|  |  |  | 1e = Emergency contraception (EC) | | 0 | 1 | 99 |
|  |  |  | 1f Condoms | | 0 | 1 | 99 |
|  |  |  | Community awareness (IEC) about benefits/location of family planning services | | 0 | 1 | 99 |
| **FOOD AND NUTRITION, WATER, NON-FOOD ITEMS** | | | | | | | |
| CMWRA21 | On average, how many meals do you have per day before the flood? | | | | 1. 0 2. One 3. Two 4. Three 5. 5. Three or more | | |
| CMWRA22 | On average, how many meals do you have per day after the flood? | | | | 1. 0 2. One 3. Two 4. Three 5. Three or more | | |
| CMWRA23 | If there is a food shortage in the house, whose food needs are prioritized? | | | | 1. Mother 2. Father 3. Boy children 4. Girl children 5. Elderly women 6. Elderly men 7. Pregnant women 8. Other, specify 9. Don’t know | | |
| CMWRA24 | If there is a food shortage in the house, who is least likely to get food so that others can? | | | | 1. Mother 2. Father 3. Boy children 4. Girl children 5. Elderly women 6. Elderly men 7. Other, specify 8. Don’t know | | |
| CMWRA25 | Do you have access to latrines and bathing facilities? | | | | 0 No  1 Yes  If No Explain : | | |
| CMWRA26 | If yes, What type of latrine/bathing facility is available? | | | |  | | |
| CMWRA27 | What are the factors affecting access to domestic water use? | | | | 1. High water price 2. Damage to the water networks 3. My community is not served by a   water network   1. Insufficient water supply, specify: 2. Other, specify: | | |
| CMWRA28 | Who usually collects food items and water for the family? (can pick up To 2) | | | | 1. Myself 2. Mother 3. Father 4. Boy children 5. Girl children 6. Elderly women 7. Elderly men 8. Other, specify 9. Don’t know | | |
| CMWRA29 | Do you have easy and reliable access to sanitary materials/napkins? | | | | 0 No  1 Yes  If No Explain : | | |
| CMWRA30 | Do you feel safe and comfortable using the latrine/bathing facility? | | | | 0 No  1 Yes  If No Explain : | | |
| **PROTECTION AND ACCESS TO JUSTICE** | | | | | | | |
| CMWRA31 | Main safety/security problems faced by women and girls | | | 1. Domestic violence specify 2. Sexual harassment 3. Being trafficked 4. Travelling alone long distances 5. Early marriage 6. Incest 7. Honour killings 8. Other, specify | | | |
| CMWRA32 | Have you faced any violence after floods | | | 0 No  1 Yes  If yes Explain : | | | |
| CMWRA33 | Where do female survivors of violence go for help? | | | 1. To their mother 2. To their father 3. Husband 4. To other family members 5. Health Clinic 6. Private clinic 7. Legal aid 8. Community leaders 9. Friends 10. International/local NGOs 11. Community centres 12. Police 13. Other, specify 14. Don’t seek help | | | |
| CMWRA34 | Where are acts of violence against women and girls most likely to occur? | | | 1. In the home 2. At schools/universities 3. In tents 4. In the shelter 5. While using the bathing facility in   the shelter   1. While travelling alone 2. While at work outside the home 3. Other, specify: | | | |
| **ACCESS TO INFORMATION AND ASSISTANCE, PARTICIPATION** | | | | | | | |
| CMWRA35 | Have you been informed of the relief and recovery assistance in a language or manner that you understand? | | | 0 No  1 Yes | | | |
| CMWRA36 | How are most women on this site getting information? | | | 1. Husband/Male 2. Member 3. Female gathering 4. Radio 5. Mosque 6. announcement 7. TV 8. Healthcare   providers   1. Newspaper 2. Camp meetings 3. van drives 4. government representative 5. Other | | | |
| CMWRA37 | Is there an effective way that you or your community can resolve problems about your current situation, including any threats to your security and ensuring everyone receives aid equitably? | | | 0 No  1 Yes | | | |
| **SUMMARY:** | | | | | | | |
| CMWRA38 | Are you satisfied with the service provided to you in this camp? | | | 1. Unsatisfied 2. Average 3. Satisfied 4. Totally Satisfied   if unsatisfied please explain | | | |
| CMWRA39 | What are the top three **barriers** to an effective response to this emergency? | | | 1.  2.  3 | | | |
| CMWRA40 | What top three factors **facilitated** the response in this emergency? | | | 1.  2.  3 | | | |
| CMWRA41 | What suggestions do you have for improving implementation in this setting? Please think about preparedness, response, and recovery. | | |  | | | |
| CMWRA42 | We talked about a lot of things today; do you have any final comments or questions? | | |  | | | |
| CMWRA43 | **COMMENTS BY INTERVIEWER:** | | | | | | |
|  | **Form status question moved here**  **End Time also moved here** | | | | | | |

| **Module 3: Currently Pregnant women** | | | | |
| --- | --- | --- | --- | --- |
| CPW1 | | What is your highest level of education | 1. No education 2. Some informal education 3. Primary (Class 1-5) 4. Secondary (Class 10) 5. College or Higher | |
| CPW2 | | What is the highest level of education of your husband | 1. No education 2. Some informal education 3. Primary (Class 1-5) 4. Secondary (Class 10) 5. College or Higher | |
| CPW3 | | Duration of Marriage | __\|__ Months/years | |
| CPW4 | | How many pregnancies have you had altogether, irrespective of whether they ended in a live birth or not? | (Incase of 0, skip to CPW7)  __\|__  (**Number of Total Pregnancies**) | |
| CPW5 | | How many of them are still alive and of them how many are sons and daughters?  (**Total number of living children)** | Sons __\|__  Daughters __\|__  Total _____ | |
| CPW6 | | How old is your youngest child? | __\|__ Months/years | |
| CPW7 | | What is your current trimester/week of pregnancy? | 1. First Trimester (0 to 13 Weeks)  2.Second Trimester (14 to 26 Weeks)  3. Third Trimester (27 to 40 Weeks) | |
| CPW8 | | How would you describe your state of health compared to before and after the flood? | 1. Increased 2. Decreased 3. Same 4. Other, specify | |
| CPW9 | | What are women's primary health problems at present? (can select 5 options)?  Do not read the list | 1. No Disease 2. Anaemia and other nutrition-related 3. ill health 4. Diarrhoea 5. Fever 6. Physical injuries 7. Disabilities 8. Bleeding 9. Miscarriage 10. Delivery related problem 11. Respiratory problem 12. Skin infection 13. Skin rashes 14. STD/HIV 15. Mental health: anxiety, distress 16. Other, specify | |
| CPW10 | | Who makes the decisions about your access to health care in the current scenario? | 1. Myself 2. My husband 3. The husband and the wife together 4. My mother 5. My father 6. My mother-in-law 7. My father-in-law 8. Other……………………… | |
| CPW11 | | What are the three most significant obstacles for women to access health care in the present scenario? | 1. Distance to the nearest healthcare facility is too far 2. Security reasons 3. No female health staff 4. Personal health issues   (disabilities, injured, etc)   1. No transportation 2. Can’t afford it 3. Can’t take time off work 4. Can’t take time away from home 5. Child-care responsibilities 6. Objection by family 7. Objection by husband 8. Other, specify: | |
| CPW12 | | Where is the first place women go to when they need health care in the current flood situation? | 1. Community workers (LHWs, CMWs) 2. Rural/community clinic 3. Health camps 4. Government clinic/public hospital 5. Midwives 6. Community centres 7. NGOs clinic 8. Drug store 9. Family 10. Traditional practitioner 11. I don’t seek healthcare 12. Private clinics 13. private hospitals 14. Other…………………………… | |
| CPW13 | | What sexual and reproductive health services have been available to you since these floods  Multiple options | 1. Information/counselling 2. Hygiene kits 3. Sanitary kits 4. Delivery kits 5. Medicines/supplies | |
| CPW14 | | From whom do you think most  women receive pre and postnatal  care in your community? | 1. Obstetrician and Gynaecologist 2. Private doctor 3. Nurse 4. Midwife 5. Medical workers/staff 6. Traditional practitioner 7. Government doctor 8. LHV 9. Community health workers 10. Other specify 11. None | |
| CPW15 | | From where are you seeking antenatal care services | 1. Community health workers 2. Rural/community clinic 3. Health camps 4. Government clinic/public hospital 5. Midwives 6. Community centres 7. NGOs clinic 8. Drug store 9. Family 10. Traditional practitioner 11. Private clinic 12. Private hospital 13. I don’t seek healthcare 14. Other…………………… | |
| CPW16 | | Where do you plan to conduct your delivery | 1. Public facility / hospital: Name 2. NGO clinic / hospital: Name 3. Private maternity clinic: Name 4. Private clinic: name 5. Private hospital: name 6. Home: Type of providers 7. Others: Specify | |
| CPW17 | | What will be the cost of delivery service you are expecting |  | |
| CPW18 | | Do you have a delivery kit distributed to you during any health camps | 0 No  1 Yes | |
| CPW19 | | After the child you are expecting now, would you like to have another child, or would you prefer not to have any more children? | 1. Want to have another child  2. No more  3. Undecided/don't know  4. Other (Specify) | |
| CPW20 | | How long would you like to wait for getting pregnant again after current pregnancy? | 1. Months  2. Years  3. Don’t know  96. Other (Specify) | |
| CPW21 | | How long do you think your husband would like to wait before getting pregnant again? | 1. Months: 2. Years:   96. Others (Specify)  98. Don’t Know | |
| CPW22 | | Do you plan to use family planning method | 0 No  1 Yes | |
| CPW23 | | If yes, which method | 1. Female Sterilization 2. Male Sterilization 3. IUCDs 4. Implants 5. Injectables 6. COC pills 7. EC pills 8. Condoms 9. Lactational Amenorrhea 10. Standard days method 11. Rhythm Method 12. Withdrawal   Other (Specify)________ | |
| **FOOD AND NUTRITION, WATER, NON-FOOD ITEMS** | | | | |
| CPW24 | On average, how many meals do you have per day before the flood? | | | 1. 0 2. One 3. Two 4. Three 5. Three or more |
| CPW25 | On average, how many meals do you have per day after the flood? | | | 1. 0 2. One 3. Two 4. Three 5. Three or more |
| CPW26 | If there is a food shortage in the house, whose food needs are prioritized? | | | 1. Mother 2. Father 3. Boy children 4. Girl children 5. Elderly women 6. Elderly men 7. Other, specify 8. Don’t know |
| CPW27 | If there is a food shortage in the house, who is least likely to get food so that others can? | | | 1. Mother 2. Father 3. Boy children 4. Girl children 5. Elderly women 6. Elderly men 7. Other, specify 8. Don’t know |
| CPW28 | Do you have access to latrines and bathing facilities? | | | 0 No  1 Yes  If No Explain : |
| CPW29 | If yes, What type of latrine/bathing facility is available? | | |  |
| CPW30 | What are the factors affecting access to domestic water use? | | | 1. High water price 2. Damage to the water networks 3. My community is not served by a   water network   1. Insufficient water supply, specify: 2. Other, specify: |
| CPW31 | Who usually collects food items and water for the family? (can pick up To 2) | | | 1. Mother 2. Father 3. Boy children 4. Girl children 5. Elderly women 6. Elderly men 7. Other, specify 8. Don’t know |
| CPW32 | Do you have easy and reliable access to sanitary materials/napkins? | | | 0 No  1 Yes  If No Explain : |
| CPW33 | Do you feel safe and comfortable using the latrine/bathing facility? | | | 0 No  1 Yes  If No Explain : |
| **PROTECTION AND ACCESS TO JUSTICE** | | | | |
| CPW34 | Main safety/security problems faced by women and girls | | 1. Domestic violence specify 2. Sexual harassment 3. Being trafficked 4. Travelling alone long distances 5. Early marriage 6. Incest 7. Honour killings 8. Other, specify | |
| CPW35 | Have you faced any violence after floods | | 0 No  1 Yes  If yes Explain : | |
| CPW36 | Where do female survivors of violence go for help? | | 1. To their mother 2. To their father 3. Husband 4. To other family members 5. Health Clinic 6. Private clinic 7. Legal aid 8. Community leaders 9. Friends 10. International/local NGOs 11. Community centres 12. Police 13. Other, specify 14. Don’t seek help | |
| CPW37 | Where are acts of violence against women and girls most likely to occur? | | 1. In the home 2. At schools/universities 3. In tents 4. In the shelter 5. While using the bathing facility in   the shelter   1. While travelling alone 2. While at work outside the home 3. Other, specify: | |
| **ACCESS TO INFORMATION AND ASSISTANCE, PARTICIPATION** | | | | |
| CPW38 | Have you been informed of the relief and recovery assistance in a language or manner that you understand? | | 0 No  1 Yes | |
| CPW39 | How are most women on this site getting information? | | 1. Husband/Male 2. Member 3. Female gathering 4. Radio 5. Mosque 6. announcement 7. TV 8. Healthcare   providers   1. Newspaper 2. Camp meetings 3. van drives 4. government representative 5. Other | |
| CPW40 | Is there an effective way that you or your community can resolve problems about your current situation, including any threats to your security and ensuring everyone receives aid equitably? | | 0 No  1 Yes | |
| **SUMMARY:** | | | | |
| CPW41 | Are you satisfied with the service provided to you in this camp? | | 1. Unsatisfied 2. Average 3. Satisfied 4. Totally Satisfied | |
| CPW42 | What are the top three **barriers** to an effective response to this emergency? | | 1.  2.  3 | |
| CPW43 | What top three factors **facilitated** the response in this emergency? | | 1.  2.  3 | |
| CPW44 | What suggestions do you have for improving implementation in this setting? Please think about preparedness, response, and recovery. | |  | |
| CPW45 | We talked about a lot of things today; do you have any final comments or questions? | |  | |
| CPW46 | **COMMENTS BY INTERVIEWER:** | | | |

Thank You

**QUALITATIVE GUIDELINES**

## In-Depth Interview Guide: Medical officer/Doctor working in the medical camp

| *SECTION I. Preliminary Information* | |
| --- | --- |
| **Was informed consent obtained**  YES ________ (Proceed)  NO ________ (STOP! Thank the participant for their time and do not proceed | |
| **Date:** | **Location of interview :** |
| **Start time:** | **End time** |

*SECTION II. Introduction*

My name is_______________. I work for _______________. We are conducting an assessment in this emergency in ______________. The purpose of this interview is to learn about health services in the humanitarian response and your experience in providing the services. During the interview, I will ask questions pertinent to the response to the flood emergency. The answers you provide will be used to inform the government and other relief organisations providing health services with the aim of addressing the challenges and impeove the access and delivery of health services. Your name will not be included in any documents or presentations, but we may include the name of this location. There is no direct benefit to you from being in this study. If you are uncomfortable with any questions during this interview you are free to stop participating at any time. You can also choose not to answer any of the questions. Please stop me at any time during the interview if you have questions or concerns. ***Is it OK to get started?***

*SECTION III. Questions*

| S. No | Questions | Responses |
| --- | --- | --- |
| **A** | **Background** |  |
|  | May I know your name (write the full name)? |  |
|  | Designation |  |
|  | Highest professional qualification |  |
|  | How old are you? | ____/____(Year) |
|  | What is your total professional experience | _____ Years |
| **B** | **Flood Impact** |  |
|  | How much flood has impacted this area/UC/District? | - To what extent was it impacted - Were the health facilities affected - To what extent the population is affected |
|  | How the flood has impacted the overall service delivery? | - Specifically for women and children - Overall health care services |
|  | Whom do you think are the most affected population groups are? | - Women - Children - Elderly - Men - Disabled |
|  | How was the overall response to the floods by the government and development partners? | - Was it immediate - Was it effective - Were efforts made to control casualties - Were you provided with any support medicine, supplies etc |
|  | What was the biggest challenge during these floods? | - Shortage of food - Shortage of medicines - Shortage of camps - Shortage of medical professionals - Any other |
|  | How regularly do you provide your caseload report or information at district level? | - Daily - Weekly - Fortnightly - Monthly - Quaterly - Semi annualy - Annualy - None |
| **C** | **Emergency Experience** | **Probes** |
|  | Was there available infrastructure to respond to health care emergencies? | - Yes - No |
|  | How many hours do the doctors work in emergency situation at camps? | - Below 6 hours - 6 to 8 hours - 8 to 12 hours - More than 12 hours |
|  | I would like to ask you about the general situation for [refugee/displaced] women in general and specifically for pregnant women | - What issues are of greatest concern among women and where do they go to receive assistance? |
|  | I would like to ask you some general questions about the delivery of sexual and reproductive health services in [location] since the floods. | - What sexual and reproductive health services were made available to the affected communities since the floods? - Tell me about the specific types of services that are available in your community. - Which organizations provide these services? |
| **D** | **Challenges during Floods** |  |
|  | In your coverage area/camp what was the biggest problem faced by the women? | - Delivery services - ANC/PNC - Family planning - General ailments - Other conditions |
|  | What are the most common symptoms the population groups are reporting with? | - Fever - Diahorrea - Cough - Etc |
|  | Did you provide services to disabaled populations. If yes, types of disabilities | - difficulty seeing - difficulty hearing - difficulty walking - difficulty remembering or concentrating - difficulty with self-care such as washing all over or dressing - difficulty communicating |
|  | Do you have adequate medical supplies to cater for the population who is visiting the camps? | - Medicines - FP Commodities - Delivery services |
|  | How these challenges /problems were met by the district government? | - How was the support provided - Supplies - Equipment/medicines - Medical camps |
|  | Were you provided with any training to cater for the situation? Specifically to provide services during floods | - Yes/no - What kind of training |
|  | Were the services free of cost provided by partners or district government |  |
|  | Have you heard about services available in your locality | - Can you tell me more about how you received this information? - How have organizations been communicating about their available services? |
|  | Overall, how do you think services within [camp/area] / during floods /any emergency could be improved? |  |
|  | *We thank you for your time. You have all helped to provide a good understanding of the situation here. Your contributions are greatly appreciated.* | |

## In-Depth Interview Guide: Community-level health care provider CMWs/LHWs

| *SECTION I. Preliminary Information* | |
| --- | --- |
| **Was informed consent obtained**  YES ________ (Proceed)  NO ________ (STOP! Thank the participant for their time and do not proceed | |
| **Date:** | **Location of interview :** |
| **Start time:** | **End time** |

*SECTION II. Introduction*

My name is_______________. I work for _______________. We are conducting an assessment in this emergency in ______________. The purpose of this interview is to learn about health services in the humanitarian response and your experience in providing the services. During the interview, I will ask questions pertinent to the response to to the flood emergency. The answers you provide will be used to inform the government and other relief organisations providing health services with the aim of addressing the challenges and impeove the access and delivery of health services. Your name will not be included in any documents or presentations, but we may include the name of this location. There is no direct benefit to you from being in this study. If you are uncomfortable with any questions during this interview you are free to stop participating at any time. You can also choose not to answer any of the questions. Please stop me at any time during the interview if you have questions or concerns. ***Is it OK to get started?***

*SECTION III. Questions*

| S. No | Questions | Responses |
| --- | --- | --- |
| **A** | **Background** |  |
|  | May I know your name (write the complete name)? |  |
|  | Respondent is a CMW or LHW | 1. CMW  2. LHW |
|  | How old are you? | ____/____(Year) |
|  | What is your total experience as CMW/LHW | _____ Years |
|  | What is the highest level of education you have achieved? | 1. No education 2. Some informal education 3. Primary (Class 1-5) 4. Secondary (Class 10) 5. Midwivery 6. College or Higher |
| **B** | **Flood Impact** |  |
|  | How much flood impacted your area? | - Was your house destroyed - The facility - Overall the region that you live in - To what extent was it impacted |
|  | How the flood has impacted the overall service delivery? | - Specifically for women and children - Overall health care services |
|  | Whom do you think are the most affected population groups | - Women - Children - Elderly - Men - Disabled |
|  | How was the overall response to the floods by the government and development partners? | - Was it immediate - Was it effective - Were efforts made to control casualties - Were you provided with any support medicine, supplies etc |
|  | What was the biggest challenge during these floods? | - Shortage of food - Shortage of medicines - Shortage of camps - Any other |
|  | What challenges have you faced during delivery of your services. | 1.  2.  3. |
| **C** | **Emergency Experience** | **Probes** |
|  | I would like to ask you about the general situation for [refugee/displaced] women in general and specifically for pregnant women | - What issues are of greatest concern among women and where do they go to receive assistance? |
|  | I would like to ask you some general questions about the delivery of sexual and reproductive health services in [location] since the floods. | - What sexual and reproductive health services were made available to the affected communities since the floods? - Tell me about the specific types of services that are available in your community. - Which organizations provide these services? |
| **D** | **Challenges during Floods** |  |
|  | In your coverage area what was the biggest problem faced by the women? | - Delivery services - ANC/PNC - Family planning - General ailments - Other conditions |
|  | How these challenges /problems were met by the district government? | - How was the support provided - Supplies - Equipment/medicines - Medical camps |
|  | Were you provided with any training to cater for the situation? Specifically to provide services during floods | - Were you provided with a delivery kit for conducting deliveries |
|  | Were the services free of cost provided by partners or district government |  |
|  | Have you heard about services available in your locality | - Can you tell me more about how you received this information? - How have organizations been communicating about their available services? |
|  | Overall, how do you think services within [camp/area] / during floods /any emergency could be improved? |  |
|  | *We thank you for your time. You have all helped to provide a good understanding of the situation here. Your contributions are greatly appreciated.* | |

##

## In-Depth Interview Guide: District level/Provincial/National level

| *SECTION I. Preliminary Information* | |
| --- | --- |
| **Was informed consent obtained**  YES ________ (Proceed)  NO ________ (STOP! Thank the participant for their time and do not proceed | |
| **Date:** | **Location of interview :** |
| **Start time:** | **End time** |

*SECTION II. Introduction*

My name is_______________. I work for _______________. We are conducting an assessment in this emergency in ______________. The purpose of this interview is to learn about health services in the humanitarian response and your experience in providing the services. During the interview, I will ask questions pertinent to the response to the flood emergency. The answers you provide will be used to inform the government and other relief organisations providing health services with the aim of addressing the challenges and improve the access and delivery of health services. Your name will not be included in any documents or presentations, but we may include the name of this location. There is no direct benefit to you from being in this study. If you are uncomfortable with any questions during this interview you are free to stop participating at any time. You can also choose not to answer any of the questions. Please stop me at any time during the interview if you have questions or concerns. ***Is it OK to get started?***

*SECTION III. Questions*

| S. No | Questions | Responses |
| --- | --- | --- |
| **A** | **Background** |  |
|  | May I know your name (write the complete name)? |  |
|  | Designation and organization |  |
|  | Highest professional qualification |  |
|  | What is your total professional experience | _____ Years |
|  | Total experience in this organization |  |
| **B** | **Flood Impact** |  |
|  | To what extent do you think floods have impacted the district/province? | - To what extent was it impacted - Were the health facilities affected - To what extent the population is affected |
|  | Was an early warning provided for the floods? |  |
|  | Before the flood were you prepared to manage such a catastrophe? | - Any plan in place - Any preparation - Any efforts to reduce the effect of floods |
|  | What measures were taken and by whom they were supported? | - Provincial Government level - District government - Partners |
|  | What coordination mechanism do you use to communicate with the higher authorities ?  (Multiple options) | - Official meeting - Telephone - Video conferencing - Emails - Official Letters - Whatsapp groups - Others: - None |
|  | By using the above mechanism, how often do you communicate with your higher authorities? | - Daily - Weekly - Fortnightly - Monthly - Quaterly - Semi annualy - Annualy - None |
|  | Do you think the coordination mechanism between the government and development partners was good? | - Support was good - Any duplication - How will you overall explain the coordination mechanism |
|  | Are you aware of the DAFPAK partners (MSS, DKT, PSI (HANDS, RSPN, UNFPA, FPAP, SRSP, Palladium, M&C Saatchi )activities in your district ? | - Support was good - Joint planning - Any duplication - How will you overall explain the coordination mechanism |
|  | How has the flood impacted the overall service delivery? | - Specifically for women and children - Overall health care services |
|  | Whom do you think are the most affected population groups are? | - Women - Children - Elderly - Men - Disabled |
|  | How was the overall response to the floods by the government and development partners? | - Was it immediate - Was it effective - Were efforts made to control casualties - Were you provided with any support medicine, supplies etc |
|  | What was the biggest challenge during these floods? | - Shortage of food - Shortage of medicines - Shortage of camps - Shortage of funds - Shortage of staff - Any other |
| **C** | **Emergency Experience** | **Probes** |
|  | I would like to ask you about the general situation for [refugee/displaced] population | - What issues are of greatest concern where do they go to receive assistance? |
|  | I would like to ask you some general questions about the delivery of sexual and reproductive health services in [location] since the floods. | - What sexual and reproductive health services were made available to the affected communities since the floods? - Tell me about the specific types of services that are available in your community. - Which organizations provide these services? |
| **D** | **Challenges during Floods** |  |
|  | How was the response to the floods managed | - Health camps - Relief camps - Food and supplies - Managing the vulnerable groups of population children, women and elderly. |
|  | Do you have adequate medical supplies to cater for the population who is visiting the camps? | - Medicines - FP Commodities - Delivery services - Referral mechanism |
|  | How were these challenges /problems met at government level | - How was the support provided - Coordination mechanisms - Supplies - Equipment/medicines - Medical camps |
|  | Did you provided any training to your staff to cater for the situation? Specifically, to provide services during floods | - Yes/no - What kind of training |
|  | Were the services free of cost provided by partners or district government |  |
|  | Did you provided any information and education material to the affected population? | - How have organizations/development partners been communicating about their available services? - What were the medium of providing information radio/tv/ teams/ etc |
|  | Overall, how do you think services within [camp/area/district/province ] / during floods /any emergency could be improved? |  |
|  | **Short- and long-term goals/ Future preparation** |  |
|  | Now the emergency is reduced, and people are moving back to their place. What is the planning and next steps |  |
|  | What are the short term and long-term goals in place that the government would like to achieve |  |
|  | Do you think you are prepared for any future catastrophe |  |
|  | What were the key lessons learned during this disaster |  |
|  | How do you plan to move forward with the lessons learned | - Do you have any emergency plan in place - Do you have plan in catering large population - Early warning - Managing the crises |
|  | Would you like to give any suggestion/recommendations |  |
|  | *We thank you for your time. You have all helped to provide a good understanding of the situation here. Your contributions are greatly appreciated.* | |

## In-Depth Interview Guide: Recently delivered women in the past 3 months

| *SECTION I. Preliminary Information* | |
| --- | --- |
| **Was informed consent obtained**  YES ________ (Proceed)  NO ________ (STOP! Thank the participant for their time and do not proceed | |
| **Date:** | **Location of interview :** |
| **Start time:** | **End time** |

*SECTION II. Introduction*

My name is_______________. I work for _______________. We are conducting an assessment in this emergency in ______________. The purpose of this interview is to learn about health services in the humanitarian response and people’s experiences in accessing these services. During the interview, I will ask questions about your experiences pertinent to your latest delivery that took place during this flood emergency. The answers you provide will be used to inform the government and other relief organisations providing health services with the aim of addressing the challenges and impeove the access and delivery of health services. Your name will not be included in any documents or presentations, but we may include the name of this location. There is no direct benefit to you from being in this study. If you are uncomfortable with any questions during this interview you are free to stop participating at any time. You can also choose not to answer any of the questions. Please stop me at any time during the interview if you have questions or concerns. ***Is it OK to get started?***

*SECTION III. Questions*

| S. No | Questions | Responses |
| --- | --- | --- |
| **A** | **Background** |  |
|  | May I know your name (write the full name)? |  |
|  | How old are you? | ____/____(Year) |
|  | What is the highest level of education you have achieved? | 1. No education 2. Some informal education 3. Primary (Class 1-5) 4. Secondary (Class 10) 5. College or Higher |
|  | What is your average household monthly income prior to the emergency? | 1. Less than 20,000 2. 20,000-50,0000 3. 50,000-70,000 4. 70,000-100,000 5. Greater than 100,000 |
| **B** | **Reproductive Health** |  |
|  | How many pregnancies have you had altogether, irrespective of whether they ended in a live birth or not? | __\|__  (**Number of Total Pregnancies**) |
|  | How many of them are still alive and of them how many are sons and daughters?  (**Total number of living children)** | Sons __\|__  Daughters __\|__  Total _____ |
|  | How old is your youngest child? | __\|__ (Months) |
| **C** | **Emergency Experience** | **Probes** |
|  | I would like to ask you about the general situation for [refugee/displaced] women in general and specifically for pregnant women | - What issues are of greatest concern among pregnant women and where do they go to receive assistance? |
|  | I would like to ask you some general questions about the delivery of sexual and reproductive health services in [location] since the floods. | - What sexual and reproductive health services have been available to you since the floods? - Tell me about the specific types of services that are available in your community. - Which organizations provide these services? |
| **D** | **Delivery Experience** |  |
|  | Before the floods were you visiting any doctor, or health service provider for antenatal care check-ups? | Ask for   - Which facility and type public or private - How was that experience |
|  | During the flood's emergency from where did you seek health care during pregnancy? | - Public or private clinic or hospital - Are there different locations you had to visit - Were the services provided nearby - Were you guided about the services from where to seek and what to do |
|  | During childbirth, from where did you seek assistance | - A trained provider - traditional birth attendants, traditional healers, midwives, etc. - Were you provided with a delivery kit - Government facility (BHU, RHC, THQ, DHQ, hospital) - Private (clinic, hospital) - Others ----------- |
|  | How was your experience | - Was it a normal delivery/caesarian section? - What was the outcome of delivery - Were you asked to follow up - What were the problems that you faced |
|  | How much do childbirth services cost? | - Are there costs of travel to get to the clinic or costs once at the clinic?) |
|  | What are your current breastfeeding practices? |  |
|  | How is your newborn baby | - Follow up visits - Vaccination/immunization |
|  | Are you visiting/seeking care for postnatal check- ups |  |
|  | Have you heard about services available in your locality | Can you tell me more about how you received this information? How have organizations been communicating about their available services? |
|  | Overall, how do you think services for pregnant women and within [camp/area] / during floods /any emergency could be improved? |  |
|  | *We thank you for your time. You have all helped to provide a good understanding of the situation here. Your contributions are greatly appreciated.* | |

## KIIs: Development Partners/Implementing Partners

| *SECTION I. Preliminary Information* | |
| --- | --- |
| **Was informed consent obtained**  YES ________ (Proceed)  NO ________ (STOP! Thank the participant for their time and do not proceed | |
| **Date:** | **Location of interview :** |
| **Start time:** | **End time** |

*SECTION II. Introduction*

My name is_______________. I work for _______________. We are conducting an assessment in this emergency in ______________. The purpose of this interview is to learn about health services in the humanitarian response and your experience in providing the services. During the interview, I will ask questions pertinent to the response to the flood emergency. . The answers you provide will be used to inform a report that might be published or presented in one or more public health forums. Your name will not be included in any documents or presentations, but we may include the name of this location. There is no direct benefit to you from being in this study. If you are uncomfortable with any questions during this interview you are free to stop participating at any time. You can also choose not to answer any of the questions. Please stop me at any time during the interview if you have questions or concerns. ***Is it OK to get started?***

*SECTION III. Questions*

| S. No | Questions | Responses |
| --- | --- | --- |
| **A** | **Background** |  |
|  | May I know your name (write the initials of the name)? |  |
|  | Designation |  |
|  | Organization |  |
|  | Highest professional qualification |  |
|  | How old are you? | ____/____(Year) |
|  | What is your total professional experience | _____ Years |
|  | Duration of experience in current organization |  |
| **B** | **Flood Impact** |  |
|  | To what extent do you think floods have overall impacted the population of Pakistan? | - To what extent was it impacted - Were the health facilities affected - To what extent the population is affected - Which province most affected - Did you expect this extent of disaster |
|  | Was an early warning provided for the floods? | - Early warning system was in place - If not why not - How long was the response phase/planning phase |
|  | Prior to floods were you prepared to manage such a catastrophe? | - Any plan in place - Any preparation - Any efforts to reduce the effect of floods |
|  | What measures were taken and by whom they were supported? | - Government level - Partners - Other funding sources |
|  | Do you think the coordination mechanism between the government and development partners was good? | - Support was good - Any duplication - How will you overall explain the coordination mechanism |
|  | How has the flood impacted the overall service delivery? | - Specifically for women and children - Overall health care services |
|  | Whom do you think are the most affected population groups are? | - Women - Children - Elderly - Men - Disabled |
|  | How was the overall response to the floods by the government and development partners? | - Was it immediate - Was it effective - Were efforts made to control casualties - Were you provided with any support medicine, supplies etc |
|  | What was the biggest challenge during these floods? | - Shortage of food - Shortage of medicines - Shortage of camps - Any other |
| **C** | **Emergency Experience** | **Probes** |
|  | I would like to ask you about the general situation for [refugee/displaced] population | - What issues are of greatest concern where do they go to receive assistance? |
|  | I would like to ask you some general questions about the delivery of sexual and reproductive health services in [location] since the floods. | - What sexual and reproductive health services were made available to the affected communities since the floods? - Tell me about the specific types of services that are available in your community. - Which organizations provide these services? |
| **D** | **Challenges during Floods** |  |
|  | How was the response to the floods managed | - Health camps - Relief camps - Food and supplies - Managing the vulnerable groups of population children, women and elderly. |
|  | Do you have adequate medical supplies to cater for the population who is visiting the camps? | - Medicines - FP Commodities - Delivery services - Referral mechanism |
|  | How were these challenges /problems were met | - How was the support provided - Supplies - Equipment/medicines - Medical camps |
|  | Did you provided any training to your staff to cater for the situation? Specifically, to provide services during floods | - Yes/no - What kind of training |
|  | Were the services free of cost provided by partners |  |
|  | Did you provide any information and education material to the affected population? | - How have organizations/development partners been communicating about their available services? - What were the medium of providing information radio/tv/ teams/ etc |
|  | Overall, how do you think services within [camp/area/district/province] / during floods /any emergency could be improved? |  |
|  | **Short- and long-term goals/ Future preparation** |  |
|  | Now the emergency is reduced, and people are moving back to their place. What is the planning and next steps |  |
|  | What are the short term and long-term goals in place that the government/partners would like to achieve |  |
|  | Do you think you are prepared for any future catastrophe |  |
|  | What were the key lessons learned during this disaster |  |
|  | How do you plan to move forward with the lessons learned | - Do you have any emergency plan in place - Do you have plan in catering large population - Early warning - Managing the crises |
|  | Would you like to give any suggestion/recommendations |  |
|  | *We thank you for your time. You have all helped to provide a good understanding of the situation here. Your contributions are greatly appreciated.* | |

1. Adapted from UNIFEM. Rapid Gender Needs Assessment of flood affected communities. 2010; [↑](#footnote-ref-2)
